# Directed Connectivity Analysis of the Brain Network in Mathematically Gifted Adolescents

**DOI:** 10.1155/2020/4209321

**Published:** 2020-08-28

**Authors:** Mengting Wei, Qingyun Wang, Xiang Jiang, Yiyun Guo, Hui Fan, Haixian Wang, Xuesong Lu

**Affiliations:** ^1^Institute of Psychology, Chinese Academy of Sciences, Beijing 100101, China; ^2^Key Laboratory of Child Development and Learning Science of Ministry of Education, School of Biological Science & Medical Engineering, Southeast University, Nanjing 210096, Jiangsu, China; ^3^Qingdao Port International Company, Ltd., Qingdao 266011, Shandong, China; ^4^Co-Innovation Center of Shandong Colleges and Universities: Future Intelligent Computing, Shandong Technology and Business University, Yantai 264005, Shandong, China; ^5^Department of Rehabilitation, Zhongda Hospital, Southeast University, Nanjing 210009, Jiangsu, China

## Abstract

The neurocognitive characteristics of mathematically gifted adolescents are characterized by highly developed functional interactions between the right hemisphere and excellent cognitive control of the prefrontal cortex, enhanced frontoparietal cortex, and posterior parietal cortex. However, it is still unclear when and how these cortical interactions occur. In this paper, we used directional coherence analysis based on Granger causality to study the interactions between the frontal brain area and the posterior brain area in the mathematical frontoparietal network system during deductive reasoning tasks. Specifically, the scalp electroencephalography (EEG) signal was first converted into a cortical dipole source signal to construct a Granger causality network over the *θ*-band and *γ*-band ranges. We constructed the binary Granger causality network at the 40 pairs of cortical nodes in the frontal lobe and parietal lobe across the *θ*-band and the *γ*-band, which were selected as regions of interest (ROI). We then used graph theory to analyze the network differences. It was found that, in the process of reasoning tasks, the frontoparietal regions of the mathematically gifted show stronger working memory information processing at the *θ*-band. Additionally, in the middle and late stages of the conclusion period, the mathematically talented individuals have less information flow in the anterior and posterior parietal regions of the brain than the normal subjects. We draw the conclusion that the mathematically gifted brain frontoparietal network appears to have more “automated” information processing during reasoning tasks.

## 1. Introduction

In neuroimaging studies, brain functional connections are described as the interrelationship of brain regions on neurophysiological activity, and the two brain regions that have similar dynamic characteristics over time provide a physiological basis for information processing and mental representation [1, 2]. In addition to functional connectivity analysis, directed connectivity analysis is another research method for connectivity analysis of brain networks. Directed connectivity can reflect the mechanism of real information processing and reveal the interactions between different brain regions. At the same time, it can assess how one brain network affects another. Previous studies have found that mathematically gifted adolescents have highly developed right hemisphere functions, interactions with the prefrontal cortex, and enhanced cognitive control between the frontal-parietal cortex and the posterior parietal cortex. The parietal-frontal integration theory (P-FIT) model explains the function and interaction of functional units in the frontoparietal network in detail [3]. White matter bundles between frontoparietal regions have fidelity, rapidity, and error-free information transmission. The temporal and occipital lobes are responsible for processing early cognitive and visual information, and the parietal cortex is responsible for receiving the results of potential sensory and intuitive processing [4]. These studies have found that the mathematical brain may reduce the implementation and supervision of the posterior parietal cortex due to the forehead, resulting in extensive activation of the posterior parietal cortex. Studies also have speculated that anterior cerebral nervous systems with mathematical talent may have a smaller effect on the posterior parietal cortex.

In the Granger causal analysis theory of directed connectivity, the lower influence and control of the anterior nervous system on the posterior parietal cortex represents a more “automated” cognitive treatment of the frontoparietal regions and a faster response to mathematical behavior. However, by summarizing the experimental data obtained from the previous practice, it can be seen that there is no significant evidence that the fast behavioral performers must be mathematically talented. Studies have shown that compared to young people in general, mathematically talented adolescents are not superior in all aspects; if they achieve higher mission accuracy, they may show delayed response time. From the results of such experimental data, the researchers speculate that, compared with other people, the posterior parietal cortex of adolescents with mathematical talent is more affected by the anterior nervous system, thus rendering their cognitive processing more “nonautomated.”

To date, most cognitive science research studies have examined the excellent cognitive control of the prefrontal cortex, enhanced frontoparietal cortex, and posterior parietal cortex; however, the exact relationship between the anterior and posterior parietal cortex in the frontoparietal regions is not known. On the other hand, since the information flow in the cortical circuit changes extremely fast, the evaluation of the influence and control relationship of this instantaneous establishment in directed connectivity analysis is still a challenging issue.

Although there are studies of mathematical brain based on functional magnetic resonance imaging (fMRI), due to the limitation of temporal resolution, it was impossible to accurately include the time information required to establish causal relationships in the brain. Therefore, electrophysiological techniques based on high temporal resolution are essential research tools for directed connectivity analysis, which will more accurately analyze the instantaneous directional information flow between frontoparietal regions.

To more accurately assess the instantaneous directional information flow between the frontoparietal regions and to more accurately establish the relationship between the influence and control between frontoparietal regions of the mathematically talented, we use Granger causal analysis based on the sliding time window to study the functional interaction between the frontal lobe and the parietal lobe during logical reasoning tasks; that is, we analyze the causal directed connectivity of brain network dynamics on the *θ*-band and *γ*-band.

## 2. Materials

### 2.1. Participants

In this study, the mathematically gifted students (*n* = 20) are selected based on their ability and performance of showing high interest in math while the other groups (*n* = 18) show high general cognitive ability but are neither specifically gifted nor interested in mathematics. The principles of selecting subjects in the mathematically gifted group are expression-based observation, mathematical academic performance, and intellectual level [5–7]. All subjects have participated in Raven's advanced reasoning test and have a math aptitude test score of greater than 32 (mean ± standard deviation: 34.6 ± 0.5). Every participant has reported that he or she has normal vision and does not take any medication for mental illness currently. They have been fully informed about the procedures of the experiments, and they provide written informed consent before data collection. Institutional Southeast University has approved this experimental protocol.

### 2.2. Experimental Design

**Figure 1:**
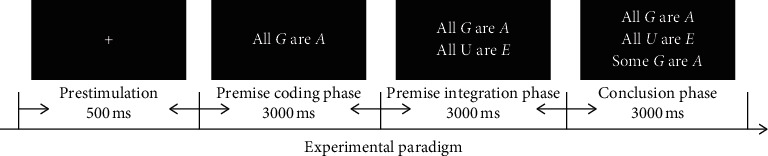
Experimental sequence design of the deductive reasoning task about word logic.

The whole experimental design consists of deductive reasoning tasks, which test the most basic mathematical thinking ability. More specifically, the blunt syllogism used in this experiment, which is a general model of deductive reasoning and is widely used in neuroscience research [8–15], consists of abstract letters. In the course of the experiment, the major premise, minor premise, and conclusion correspond to the premise coding phase, premise integration phase, and conclusion phase, respectively. They are presented on the computer screen one by one in a process which lasts for 25–30 minutes for each subject (see Figure 1).

When a conclusion sentence appears on the screen, the participant is asked to judge whether the conclusion sentence is consistent with his own inference, and the subject is required to press the keyboard to indicate his decision within a limited time of 3000 ms. The entire experimental stimulus is presented by the E-Prime 2.0 software.

### 2.3. Data Preprocessing

Data were bandpass filtered between 1 Hz and 60 Hz and baseline-corrected by subtracting the average of the first 1000 ms of the baseline period. Independent component analysis (ICA) in EEGLab was used to remove the eye blinking artifacts [16, 17]. Finally, during the deductive mathematical task, 780 trials were maintained with 400 trials of the mathematically gifted group and 380 retained trials of the control group.

### 2.4. Cortical Dipole Source Signal Conversion

In brain connectivity study, neuroelectric signals taken directly from the scalp electrodes are affected by volume conduction, which leads to overestimation of the coherence between the source signals during data analysis. The EEG signal collected by the scalp is the result of superposition of multiple source signals at the electrode points. So, the electrode points that in fact are not connected will possibly produce false connectivity, which will affect the correct evaluation of brain network connectivity. In fact, to overcome the phenomenon of volume conduction, this study conducts the EEG source localization of the collected EEG signals to further analyze the connectivity of the brain network in the cortical layer. The reconstruction process for the cortical dipole source signal is to convert the original EEG time series into a time series of cortical dipole source signals of the same length of time without losing the original time resolution of the EEG signal. In this study, the source current estimation method provided by the open-source Brainstorm neural signal processing tool is used to perform source space conversion on EEG signals. The reconstruction process of cortical dipole source signal includes the calculation of forward model and inverse model.

The first step of reconstructing the cortical dipole source signal is to calculate the forward model, which is essential to explain how the source signal flowing on the cortical surface affects and generates the scalp electrode signal. In this study, the ICBM152 magnetic resonance imaging template recognized by the International Brain Atlas Union is used to calculate the location coordinates of the 60 EEG electrodes on the template. At the same time, we use the symmetrical boundary element method to calculate the electrical properties of the head so as to obtain the volume conduction model of the head. The forward model calculates a head model matrix which is called the forward model matrix. Its size is the number of electrodes on the scalp and the number of dipoles on the cortical surface. Because the EEG signal has the highest signal-to-noise ratio and sensitivity in the cerebral cortex, the source space is defined in the cerebral cortex and is dispersed into 15002 grids as a group of equivalent current dipole sources. The number of electrode channels used in this study is 60. Consequently, the positive model matrix of dimension 60 by 15002, also known as the guide matrix, is obtained by the above process. Next, by calculating the noise covariance matrix of the baseline time region signal, we remove the sensor noise in the signal to reduce the impact on the inverse model calculation.

**Figure 2:**
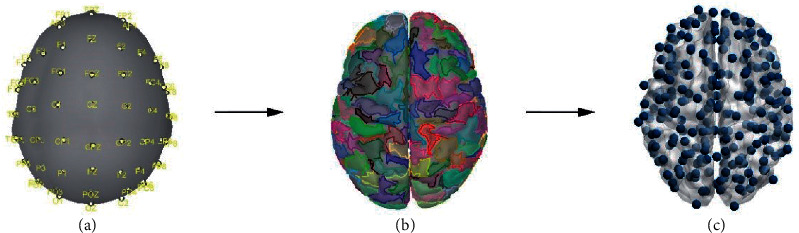
Head model, brain segmentation, and cerebral cortex dipole. (a) Standard head model and spatial distribution of 60 EEG electrodes on the scalp. (b) The cortical surface is segmented into 256 anatomical regions of interest (128 regions per hemisphere) based on a random homogeneous segmentation method. (c) Distribution of network nodes of 256 cortical layer regions after downsampling.

The calculation of the inverse model uses the forward model and the current density map, which generate the inverse kernel matrix whose dimension is the number of scalp electrodes on the cortical surface. The inverse kernel matrix is multiplied by the original EEG scalp electrode signal matrix to obtain the dipole current source located on the surface of the cortex. In this study, based on the random homogeneous segmentation method provided by the Brainstorm toolkit, the cortical surface is divided into 256 regions of interest, and each region is downsampled to a cortical dot by fast principal component analysis, which is used as the network node of the cortical region. These 256 distributed cortical vertices will be used as basic network nodes for the subsequent brain network connectivity analysis (see Figure 2).

## 3. Methods

The Granger causality theory was first proposed by Wiener [18]. Clive Granger, an economist and Nobel laureate in economics, further developed linear vector regression causal relationship model based on random time series data [19]. First developed in the context of the economic theory of measurement, Granger causality analysis has a wide range of applications in economics, climate science, and neuroscience. In this paper, we used the directional coherence analysis based on Granger causality to study the interactions between the frontal brain area and the posterior brain area in the mathematical frontoparietal network system during the deductive reasoning task. The directional coherence described by Baccala et al. [20] is a frequency domain description of Granger causality between multivariate time series represented by vector autoregressive models. It was generalized further as partial directed coherence (PDC) [21] and then the normalized PDC [22]. All versions of the directed coherence are linear. The modern nonlinear Granger causality is rather a different measure. The most relevant versions with different nonlinear functions can be found in [23–26]. Moreover, the versions for nonstationary data are described in [27–29]. In this study, we used the directional coherence analysis based on Granger causality to build causative brain network.

The Granger causality value between the variable *y* and the variable *x* is calculated as follows:

(1) $$\begin{array}{c} f_{Y⟶X\;}\left(\omega \right)=-\ln\left(1-\frac{H_{XY}\left(\omega \right)\sum_{\left.Y\right|X}H_{XY}^{*}\left(\omega \right)}{\left|S_{XX\;}\left(\omega \right)\right|}\right), \end{array}$$

where the partial covariance matrix *Σy*|*x* is defined by

(2) $$\begin{array}{c} \Sigma_{\left.Y\right|X}=\Sigma_{YY}-\Sigma_{YX}\Sigma_{XX}^{-1}\Sigma_{XY}. \end{array}$$

In equation (1), *S* stands for the estimated cross-power spectral density (CPSD) matrix and Σ for the residuals covariance matrices. For a VAR process, the CPSD admits unique spectral factorization [20]:

(3) $$\begin{array}{c} \;S\left(\omega \right)=H\left(\omega \right)\Sigma H^{*}\left(\omega \right), \end{array}$$

where transfer function *H*(*λ*) is defined as the inverse of the Fourier transform of the regression coefficients:

(4) $$\begin{array}{c} \;H\left(\lambda \omega \right)={\left(I-\sum_{k=1}^{p}A_{k}e^{-ik\omega }\right)}^{-1},\;0\leq \omega \leq 2\pi . \end{array}$$

Granger causality cannot be processed with nonstationary data, which is the disadvantage of Granger causality, while most of the EEG neurophysiological data are nonstationary and typical randomness. Therefore, we need to process the EEG data, first of all, detrending and averaging the data to avoid excessive linearity of the data. Then, the ADF test or KPSS test is carried out on the data. Next, for the test-passed data, the autoregressive model order is obtained by using the BIC criterion, and for, the untested data, the autoregressive model order is obtained by using the BIC criterion after the first-order difference. Then, the directional coherence analysis based on the Granger causality model is used to quantify the causal relationship between the channels to generate the adjacency matrix of the causality brain network.

Due to the instability of EEG data, the deductive reasoning task cycle is divided into 90 time windows with length of 100 ms. The Granger causality based on the sliding time windows is used to analyze the oscillation between brain network regions in the process of deductive reasoning tasks. Among them, the sliding time window divides the data into nonoverlapping windows, and the window whose logic is shorter is more likely to be approximately static, which means that there is a tradeoff between the possibility of stationarity (the shorter the better) and the accuracy of the model fitting (the longer the better).

In this study, 40 pairs of cortical nodes in the frontal lobe and parietal lobe are selected as regions of interest (ROIs), which construct a binary causal directional connection network. The connection edges are obtained by comparing significant differences from baseline GC values. More precisely, when comparing the GC values during the task with the GC values of baseline period at each sliding window, if the GC value has a significant difference, the connection between the pair of nodes is set to 1; otherwise, it is set to 0.

At each sliding window, we calculate the characteristic path length of each resulting network. For a global network, the characteristic path length refers to the average minimum number of connected edges that pass between one node and another in the network. The short characteristic path length in the network represents higher parallel information transfer capability and higher network global performance. The formula for calculating the length of the characteristic path is

(5) $$\begin{array}{c} L=\;\frac{1}{n}\underset{i\in N}{\sum }L_{i}=\;\frac{1}{n}\underset{i\in N}{\sum }\frac{\sum_{j\in N,j\neq i}m_{ij}}{n-1}, \end{array}$$

where *L*_*i*_ represents the average distance between node *i* and other nodes in the network and *m*_*ij*_ represents the shortest path between nodes *i* and *j*.

Causal density: causal density is expressed as a parameter to measure the degree of close relationship between network nodes. The higher the causal density value, the closer the relationship between the network nodes. The formula for calculating the causal density of the network is as follows:

(6) $$\begin{array}{c} CD\left(X\right)=\frac{1}{n\left(n-1\right)}\sum_{i\neq j}F_{x_{i}⟶x_{j}\left|x_{\left|ij\right|}\right.}, \end{array}$$

where *x*_|*ij*|_ is the *X* network after removing *x*_*i*_ and *x*_*j*_.

Causal density is used to describe the density of connected edges between nodes in a causal network, which is calculated according to the value of Granger causality in the network. On the other hand, the characteristic path length, which calculates the average value of the shortest path between two points in the binary directed connection network, is also based on the Granger causality value. The difference, however, is that the connection edges of the network are compared with the Granger causality value of the baseline period to form binary causal network in computing the characteristic path length. Both the causal density and the characteristic path length use Granger causality value but from different dimensions. The former uses causality value directly, and the latter is a graph theory analysis value based on binary causal network.

## 4. Data Analysis

### 4.1. Statistical Test

In this study, ANOVA statistical test is used to analyze the significant differences in the causal density of directed connectivity networks between mathematical talent subjects and ordinary subjects in each time window. To evaluate the significance of Granger causality in the task period and the baseline period, this experiment uses a cluster-based permutation algorithm to solve the problem of multiple comparisons in the experimental process, which generates a permutation ordering of 200 raw Granger causal data sets [30].

### 4.2. Behavioral Performance

As shown in Table 1, compared with the control group, the raven advanced reasoning test scores of the mathematically gifted group are significantly higher and have significant differences.

Additionally, in the behavioral performance of the deductive reasoning task, compared with the ordinary subjects, the mathematically talented individuals show a higher task response accuracy rate of 79.8% ± 11.6% compared to the control group with 62.95% ± 13.655% (mean ± standard deviation). At the same time, the reaction time (the sum of the trial time from the gaze point to the time that the button is pressed) also shows significant intergroup differences. The mathematically talented group shows a shorter response time of 168358.90 ± 34348.17 ms compared to the control group with 231370.85 ± 50932.80 ms (mean ± standard deviation).

### 4.3. Granger Causal Connection Network

**Figure 3:**
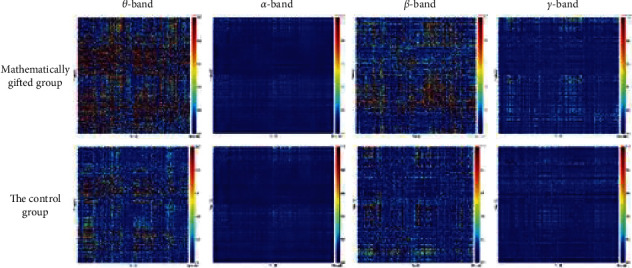
Spatial distribution map of the average Granger causality in the *θ*-band, *α*-band, *β*-band, and *γ*-band.

The spatial distribution map of average Granger causality in *θ*-band, *α*-band, *β*-band, and *γ*-band is illustrated in Figure 3. We find that the *θ*-band causal network has a higher Granger causality value than the *γ*-band.

**Figure 4:**
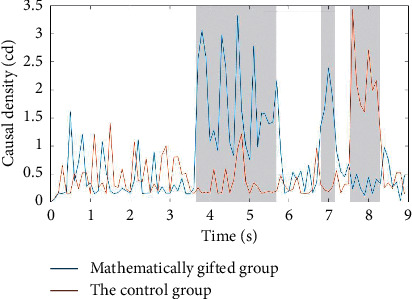
Mathematically gifted adolescents and the control group causal density map in the *θ*-band. The gray background area is represented as a time zone in which the mathematically gifted adolescents and the control group have significant differences in the causal density.

As shown in Figure 4, in the deductive reasoning process, there are significant differences between the mathematically gifted adolescents and the general subjects in the premise integration phase and the conclusion phase. In the premise integration phase and the middle and early stages of the conclusion, the mathematically gifted adolescents show higher causal density than the control group. In the middle and late stages of the conclusion phase, the mathematically gifted adolescents show lower causal density than the control group.

Based on the above research results and the behavioral performance of the participants, we speculate that in the conclusion stage of the inference task, the mathematically gifted adolescents show more efficient and rapid causal mobility than the average subjects. At the same time, in the premise integration stage of the reasoning task, the mathematically gifted subjects show higher node tightness than the average subjects for a longer period of time. This indicates that in the premise integration stage, the mathematically gifted adolescents are higher than the common subject cortex vertices.

**Figure 5:**
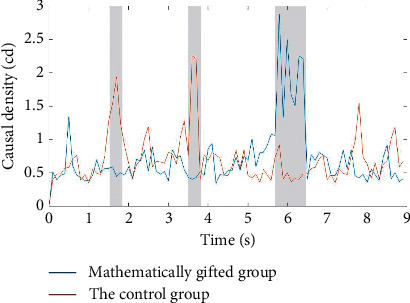
Mathematically gifted adolescents and the control group causal density map in the *γ*-band. The gray background area is represented as a time zone in which the mathematically gifted adolescents and the control group have significant differences in the causal density.

As shown in Figure 5, compared with the *θ*-band, there are significant differences in the premise coding, premise integration phase, and conclusion phase in the *γ*-band between the mathematically gifted adolescents and the ordinary subjects. In the early stage of the premise coding and the premise integration phase, the mathematically gifted adolescents show lower causal density values than the control group. In the early stage of the conclusion phase, the mathematically gifted adolescents show higher causal density values. We speculate that in the early stage of premise coding and premise integration, ordinary subjects show higher causal mobility than those of mathematically talented individuals. In the early stage of the conclusion phase, mathematically talented subjects show higher causal mobility.

According to Tables 2 and 3, the causal density values in the *θ*-band are significantly higher than the causal density values in the *γ*-band in the premise coding stage and the premise integration stage. We speculate that, in the early stages of the entire inference task process, subjects show higher causal flows between nodes in the *θ*-band.

**Figure 6:**
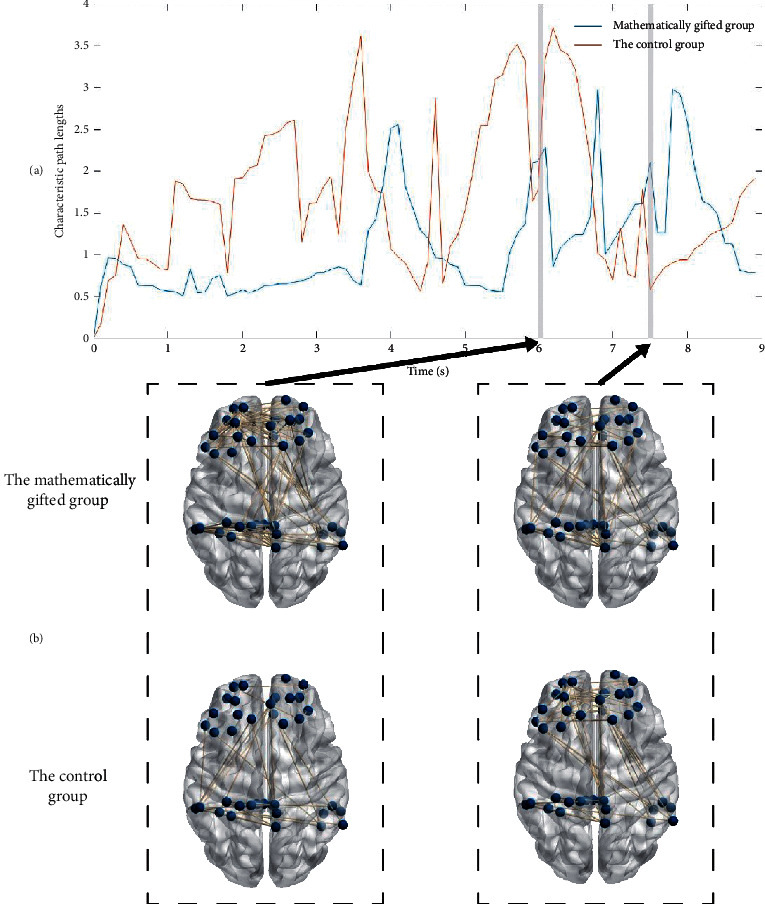
(a) Distribution of characteristic path lengths of the frontoparietal network in the *θ*-band during the deductive reasoning task. (b) During the deductive reasoning task time window (6000 ms–6100 ms, 7500 ms–7600 ms) in the *θ*-band, the effective connection network maps between the mathematically gifted group and the control group.

**Figure 7:**
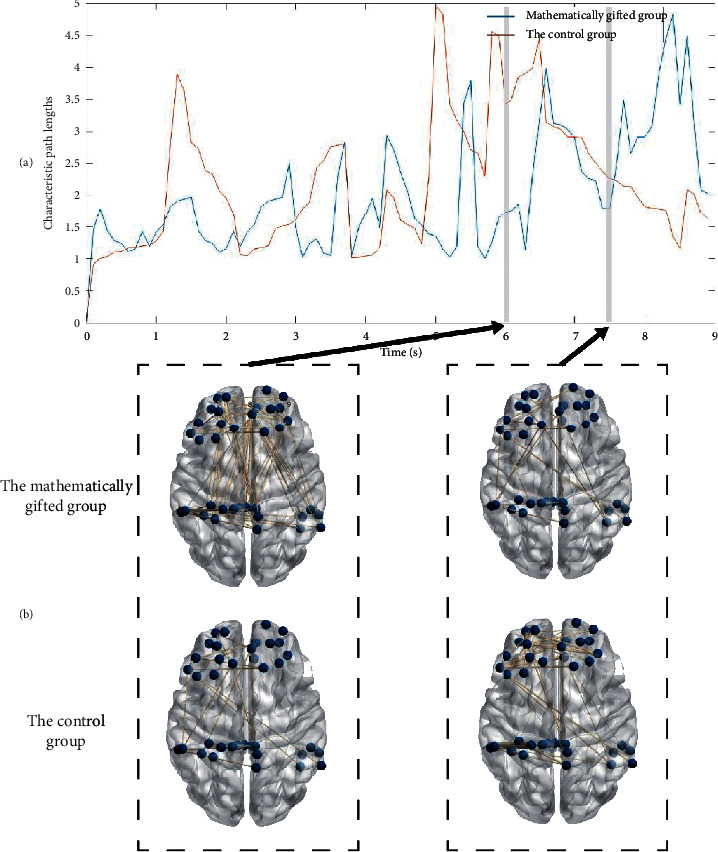
(a) Distribution of characteristic path lengths of the frontoparietal network in the *γ*-band during the deductive reasoning task. (b) During the deductive reasoning task time window (6000 ms–6100 ms, 7500 ms–7600 ms) in the *γ*-band, the effective connection network maps between the mathematically gifted group and the control group.

Figures 6(a) and 7(a) depict the evolution of the characteristic path length of the GC networks for each group type in the *θ*-band and the *γ*-band. As shown in Figure 6(a), the mathematically gifted group has a lower characteristic path length distribution than the control group by the distribution map of the characteristic path length in the *θ*-band. Of note in this figure, the characteristic path length of the mathematically gifted group during the 7000 ms–9000 ms window is significantly higher than that of the control group. Since research has pointed out that the characteristic path length may reflect brain's ability to conduct parallel information processing and the mathematically talented subjects' right reaction time is significantly less than the ordinary subjects, we hypothesize that the mathematically talented group is not in the task processing during this time period. That is, the mathematically talented subjects have completed the logical reasoning task early and have been converted into the default working mode.

By comparing the characteristic path lengths of the two frequency bands, we see that the *θ*-band causal connection network has a shorter characteristic path length than the *γ*-band causal connection network. Since the characteristic path lengths can be expressed as the network having higher parallel information transmission capability and higher global efficiency, we conclude that the network in the *θ*-band has higher flatness information transmission capability and higher network global efficiency.

At the time window, where the characteristic path lengths of the mathematically gifted group and the control group change significantly, a causal connection snapshot is performed on the binary causal effective connection network, as shown in Figures 6 and 7. From the snapshot (Figures 6(b) and 7(b)), we see that the mathematically gifted group shows a stronger network connection than the control group during the time window from 6000 ms to 6100 ms. Simultaneously, in the 7500 ms–7600 ms time window, the mathematically talented individuals show less directed connectivity between the right prefrontal and posterior parietal regions than the normal subjects.

According to Tables 4 and 5, it is found that the characteristic path length of the *θ*-band in the mathematically gifted group is significantly lower than the characteristic path length in the *γ*-band throughout the inference task phase. However, for the control group, only in the conclusion phase, the characteristic path length of the *θ*-band is significantly lower than the characteristic path length of the *γ*-band.

## 5. Discussion

In causal network analysis, the causal density value is used as an important parameter to measure the tightness of the connection between nodes of the causal network. In the early stage of the conclusion of the inference task, the mathematically talented individuals show higher causal density values than the control group, indicating a close connection causal relationship. At the same time, combined with the global neuron work area theory [31, 32], in the decision-making stage—that is, when the mathematics talents select the problem results—the mathematically talented brain is very closely linked, and it will be originally separated. The unconscious processor is recruited through the efficient integration of the nervous system to solve new problems, and this analysis is consistent with previous research findings.

Additionally, causal flow values are used as parameters to evaluate the causal effects of neuronal information processing and quantify the importance of different nodes in the entire causal network. In the process of reasoning, the cerebral ventricles in the mathematical talents show more causal flow growth and the posterior parietal cortex shows a reduced causal flow value. Therefore, there is an enhanced forehead network causal flow in the reasoning process of the mathematically talented adolescents, which is characterized by enhanced central function of the anterior nervous system and lower causal flow in the posterior parietal cortex. We believe that the anterior brain region acts as an excellent central executive processor. It affects the posterior parietal cortex and promotes brain function integration during problem-solving.

Based on the above results, we speculate that in the middle and late stages of the conclusion stage, the mathematically talented subjects have less information flow in the anterior and posterior parietal regions of the brain than the normal subjects. More specifically, in the middle and late stages of the conclusion phase, the anterior nervous system of the brain of the mathematically talented exerts a lower influence on the posterior parietal cortex. Therefore, the frontoparietal regions are characterized by automated information processing and faster response time. This conclusion is consistent with the early fMRI study that the implementation and supervision of the reduction in forehead result in a posterior parietal cortex that is extensively activated by the brain. It is hypothesized that the anterior nervous system exerts a lower effect on the posterior parietal cortex. At the same time, the lower influence and control of this anterior nervous system on the posterior parietal cortex can be considered as a more “automated” cognitive treatment of the frontoparietal regions.

On the other hand, the results of this study show that the *θ*-band frontoparietal regions have higher flatness information transmission capability and higher network global efficiency [33, 34]. The early studies about EEG have pointed out that event-related *θ*-band oscillations are associated with brain cognitive load and memory performance, and the prefrontal cortex is a working memory-related brain region. Moreover, this research experiment requires the participants to focus on keeping the consciousness in the task processing state, continuous information processing and integration, accessing information in working memory, and finally presenting conclusions and making comparisons and choices with their own judgments. Therefore, we conclude that, during reasoning tasks, the frontoparietal regions of the mathematically gifted individuals show stronger working memory information processing in the *θ*-band.

## 6. Conclusion

This paper employs the directional coherence analysis based on Granger causality to analyze the correlation of the directed connectivity of the brain network in the *θ*-band and the *γ*-band. A binary effective causal connection network is constructed for the cortical source signal in the frontoparietal regions, and the difference of the connection networks between the mathematically talented individuals and the ordinary subjects is analyzed by graph theory. The real-time information processing frontal and posterior parietal network for reasoning tasks has practical significance for the development and utilization of cortical resources in the mathematical learning of children and adolescents.

## Figures and Tables

**Table 1:** Behavioral differences between groups in deductive reasoning tasks.

	Mathematically gifted group	Control group	*p*
Raven advanced reasoning test score	34.6 ± 0.5	22.8 ± 0.4	^*∗∗*^
Response correct rate	79.8% ± 11.6%	62.95% ± 13.655%	^*∗∗*^
Reaction time	168358.9 ± 34348.17 ms	231370.85 ± 50932.80 ms	^*∗∗*^

*p*: significance level, ^*∗*^indicates *p* < 0.05, ^*∗∗*^indicates *p* < 0.01.

**Table 2:** Significant differences in the causal density of the mathematically gifted group in different frequency bands in the deductive reasoning task

Causal density	*θ* − band	*γ* − band	*p*
Premise coding phase	0.56 ± 0.19	0.40 ± 0.29	^*∗*^
Premise integration phase	0.83 ± 0.56	1.33 ± 0.98	^*∗∗*^
Conclusion phase	0.75 ± 0.51	0.61 ± 0.55	0.317

*p*: significance level, ^*∗*^indicates *p* < 0.05, ^*∗∗*^indicates *p* < 0.01, ^*∗∗∗*^indicates *p* < 0.001.

**Table 3:** Significant differences in the causal density of the control group in different frequency bands in the deductive reasoning task.

Causal density	*θ* − band	*γ* − band	*p*
Premise coding phase	0.75 ± 0.37	0.45 ± 0.35	^*∗∗*^
Premise integration phase	0.75 ± 0.46	0.37 ± 0.27	^*∗∗∗*^
Conclusion phase	0.64 ± 0.26	0.80 ± 0.56	0.347

*p*: significance level, ^*∗*^indicates *p* < 0.05, ^*∗∗*^indicates *p* < 0.01, ^*∗∗∗*^indicates *p* < 0.001.

**Table 4:** Significant differences in the characteristic path lengths of the mathematically gifted group in different frequency bands in the deductive reasoning task.

Characteristic path length	*θ* − band	*γ* − band	*p*
Premise coding phase	0.66 ± 0.12	1.55 ± 0.35	^*∗∗∗*^
Premise integration phase	1.13 ± 0.56	1.72 ± 0.77	^*∗∗∗*^
Conclusion phase	1.56 ± 0.65	2.82 ± 0.90	^*∗∗∗*^

*p*: significance level, ^*∗*^indicates *p* < 0.05, ^*∗∗*^indicates *p* < 0.01, ^*∗∗∗*^indicates *p* < 0.001.

**Table 5:** Significant differences in the characteristic path lengths of the control group in different frequency bands in the deductive reasoning task.

Characteristic path length	*θ* − band	*γ* − band	*p*
Premise coding phase	1.55 ± 0.65	1.69 ± 0.80	0.467
Premise integration phase	1.97 ± 0.95	2.41 ± 1.15	0.113
Conclusion phase	1.62 ± 0.95	2.55 ± 0.84	^*∗∗∗*^

*p*: significance level, ^*∗*^indicates *p* < 0.05, ^*∗∗*^indicates *p* < 0.01, ^*∗∗∗*^indicates *p* < 0.001.

## Data Availability

All data included in this study are available upon request by contact with the corresponding author.
